# The Institutional Library

**Published:** 1919-08-02

**Authors:** 


### THE INSTITUTIONAL LIBRARY.

**On Gunshot Injuries to the Blood Vessels.** By George Henry Makins, G.C.M.G., C.B., President of the Royal College of Surgeons of England. Published in Bristol by John Wright and Son, Ltd., in London by Simpkin, Marshall, Hamilton, Kent and Co., Ltd. 21s. net.

This volume of some 250 pages is founded upon experience gained in France during the war periods 1914-1918, when the author was Consulting Surgeon to the British Expeditionary Force. It describes essentially the individual practical experience of one surgeon who, as he states in his preface, has made no attempt to deal with the literature of the subject. Coming from so eminent a man, such a record is invaluable, not merely for the excellent and interesting descriptions of traumatic cases which he provides, but for the conclusions he has drawn from innumerable observations, both large and small. The subject of wounds implicating the largest vascular trunks has been considered so frequently in the past, and such special interest has attached to the treatment of those caused by projectiles that it is not surprising that the general character and behaviour of this type of injury have undergone no material change. In his opening chapter, however, the author points out that their special features, consisting in the tendency to spontaneous cessation of primary hæmorrhage even in the presence of very extensive damage to the vessel involved; the frequency with which such forms of injury are the occasion of secondary hæmorrhages; and the large proportion of these which are followed by the development of one of the several forms of traumatic aneurysm, have been maintained in type, although the much greater experience provided by the vast scale of modern fighting has given us unique opportunity to refine our knowledge. This part of the subject is dealt with in highly interesting fashion, and a chapter on frequency with which individual vessels are implicated, determining factors and the variety of wounds met with is excellent, and details tabulated in a way sufficiently clear to serve as a perfect introduction to the more specialised regional descriptions which follow. The volume is well and profusely illustrated, and the general production good; but what is, perhaps, most valuable to those who must learn of the war and its surgical lessons from the writings of those to whom knowledge and skill brought a deserved reward of first-hand experience, is found in the happy combination of the unique, the interesting and the practically instructive which the author has provided.

**Vicious Circles in Disease.** By Jamieson B. Hurry, M.A., M.D. (Cantab). (London : J. and A. Churchill. 15s. net. Third edition.)

Eight years have elapsed since the publication of the first edition of the monograph, and during this time the author has found a careful review of the subject necessary, with the result that nine fresh chapters have been added, and a large part of the work rewritten. A welcome addition to the setting forth of a view which, though interesting in the abstract, was somewhat discouraging to those whose duty it was to cure as much as to study disease, is the space allotted to methods of "breaking the circles," an aspect of therapeutics which may, as the author asserts, have received little attention in the past, but which nevertheless is the essential requirement if we accept these ideas on disease.

**The Newer Knowledge of Nutrition.** By E. V. McCollum. (New York : The Macmillan Co. Pp. 199. Price 6s. 6d. net.)

As the author of this monograph quite rightly points out, the enormous wastage, material and human, of the war has rendered it more important than ever that knowledge of the principles of nutrition shall be correctly and widely disseminated. The demoralisation of agriculture over wide areas, together with the shortage of tonnage for the transportation of food, have reduced the food supply of Europe to the danger point, and have cut off in great measure the opportunity for securing the variety which exists in normal times. It is bare justice to say that the author has done a sound piece of work in presenting in a form easily comprehended by the masses a *résumé* of the latest researches upon nutrition, many of which will be new to the medical profession on this side of the Atlantic, let alone to the general public. It is a pleasure to recommend a book in which sound commonsense is displayed along with expert scientific knowledge, and in which also both are exhibited to the public in a manner which must convince even the crassest sentimentalist of the necessity for animal experimentation if progress in human pathology and therapeutics is to be maintained. It is a pity his publishers have not seen their way to put it on the market at a smaller cost : they would surely have been rewarded by a much larger circulation.

**Theory and Practice of Massage.** By Beatrice M. Goodall-Copestake, Examiner to the Incorporated Society of Trained Masseuses; Teacher of Massage and Swedish Remedial Exercises to the Nursing Staff of the London Hospital. Second Edition. (London : H. K. Lewis. Price 9s.).

The second edition of the "Theory and Practice of Massage," with its important additions, ranks as a premier work of inestimable practical value. Devoid of abstruse technicalities, the use and nature of appliances are fully expounded. An appendix of specimen examination questions, and a glossary of technical terms and their meanings terminate and complete the work. Practical hints and advice are given to prospective students upon choosing the very best training, and the extent of training desirable. The practice of massage, and its incorporation into medical science and practice, is traced from its early beginnings to the place it now occupies as an important anxiliary to all branches of medicine.

**Bydand: Poems of War and Peace.** By John Mitchell. With an introduction and notes by J. M. Bulloch. Aberdeen: William Smith and Sons, The Bon-Accord Press, 1918.

We are indebted for this volume to the matron of a Scotch Military Hospital, who warned us in a covering letter that most of the poems were written in broad Scots. In the introduction Mr. Bulloch tells us that the author is "a plain business man," whose "verse is a genuine and irrepressible expression of himself, his origins, and his early environment; and its sincerity appeals to all who know the vigorous vernacular"—the art of writing verse in which he very properly defends. Most of the poems have the qualities which are associated with ballads—an easy flow, a thread of narrative, a homely simplicity of style. The verse maintains its level and neither rises above, nor falls below, its natural plane. For southern readers quotations must be confined to poems not

**The Institutional Library**—(*continued*).

written in Scots, and a characteristic example may be taken from "The Poet":

"He sings the ocean's changing moods
The ordered harmony o' heaven;
Ower life's complexity he broods,
Invoking light which ne'er is given.
He sings the deathless deeds o' fame
On land and sea through ages down;
An' roon ilk' honoured hero's-name
He weaves sweet garlan's o' renown."

The verse is so steady that one hardly notices at first the bankruptcy of the fourth line. Yet compare this empty verse with the great one of the English poet who said the poet's function was that "of superseding faith by sight," and the quality of the work begins to be measured.

**The Physiological Feeding of Children.** By ERIC PRITCHARD, M.D. (London: Henry Kimpton. 1919. Pp. 59. Price 3s. 6d. net.)

IN this well-printed paper-backed monograph Dr. Pritchard propounds the principles of dietetics as they apply to children above the age of twelve months. In spite of a passion for "calories," which will, it is to be feared, make a good many doctors and nurses, as well as parents, regard him as an amiable crank, there is a vast amount of sound sense in his contentions. He is very far from being a mere theorist, divorced from practicable expediency; and his teaching, if iconoclastic in some respects, will probably stand the test of time and experience. He is, of course, up against the most intense and heart-breaking conservatism innate in the human species, nowhere more so than in these islands—the mentality which cloaks ignorance and unwillingness to learn by the hoary but still formidable formula beginning, "What was good enough for my grandfather . . ."

**Anæsthetics, and the Nurses' Duties.** By A. DE PRENDERVILLE, LL.B., M.R.C.S. (London: William Heinmann. Price 3s. 6d.).

SPECIAL emphasis is given to the nurse's duties in relation to her assistance in the administration of anæsthetics and to the anæsthetist. Points are enlarged upon for guidance and help in a duty that claims the particular qualities of conscientiousness and presence of mind. No point is omitted that has any bearing upon the administration or the condition of post-anæsthesia; the importance of such knowledge should not fail to be added to the training of nurses. In this work a nurse seeking enlargement of her sphere will find interesting and practical advice, explicit and simple in text, easy of understanding, with few technicalities to confuse the mind.

**Technique of the Irrigation Treatment of Wounds by the Carrel Method.** By J. DUMAS and ANNE CARREL. Authorised translation by ADRIAN V. S. LAMBERT, M.D. (London: William Heinemann. 1918. Pp. 77. Price 6s.)

THE wonderful surgical Clinique for the treatment of wounds by hypochlorite solution, at Compiègne, is well known to large numbers of surgeons of the allied nations who have been serving on the Western Front during the war. Many of these are indebted to Dr. Carrel, its founder and chief surgeon, for his unselfish hospitality in placing his experience and the clinical resources of his hospital at their disposal. Dr. Carrel's professional brethren are now further indebted to him for the publication of the technic of his method. The instructions are very clearly expressed, no detail necessary to ensure the success of the inexperienced has been omitted: the preparation, testing, and care, of the solution are described, and the means of application by ampoule, rubber instillation tubes, and glass distributors, are rendered "fool proof" by the addition of plates which are veritable works of art. The technic for the control of the method by microscopical examination of the discharges is also very clearly indicated, including the interpretation of the results. The success of the process depends upon the meticulous observance of details: when properly carried out, its results, in extensive lacerated and deeply infected wounds, are indisputable. The book is clearly printed on very good paper, and is translated in the attractive style of the authors.

**Barbed-Wire Disease.** By A. L. Vischer, M.D. Translated, with an introductory chapter, by S. A. K. Wilson, M.D., F.R.C.P. (London: J. Bale, Sons and Danielsson, 1919. Pp. 84. Price 3s. 6d. net.)

The author of this monograph, a Swiss doctor, had ample opportunities in his neutral country of observing the effects of war imprisonment on the mentality both of soldiers and civilians, and in Germans, French, and English. Also, though he is apparently afraid to publish his observations, he must have accumulated plenty of evidence bearing on the treatment of their captives by the principal belligerents. His notes are interesting and instructive, though we think it a pity that he should use the unscientific and unnecessary term "Barbed-Wire Disease" to describe certain varieties of neurasthenia. Incidentally, there is far more of common sense and illumination of the subject in Dr. Wilson's introductory chapter than in the whole of the rest of the book. It is impossible to avoid a certain amount of disappointment that the psycho-neuroses of war captives have not been more satisfactorily catalogued, though admittedly there is a fund of useful information on this topic in Dr. Vischer's pages.

**Catalogue of Lewis's Medical and Scientific Circulating Library.** (H. R. Lewis and Co. 12s. 6d. Revised to end of 1917.)

WE welcome the appearance of the new edition of Lewis's Library Catalogue (1918), and of which the previous edition appeared in 1908—though since that date it has been necessary to issue two supplements, one for the period 1908-10 and one for 1911-13.

The catalogue as it now appears in its entirety is clearly printed, but in smaller type than formerly—thus much economising space. The present edition is revised up to the end of 1917, and consists of 498 pages. The first part is an alphabetical list of authors, with the titles of their books, the dates of publication and the publishing prices. This part must comprise nearly 18,000 titles, while the second part is a classified list of subjects with some 13,000 references, giving the names of authors who have written thereon, and for the full title it is only necessary to refer to the front of the book. The indication of the published price of each book is a very great advantage, and one has only to turn up their particular requirement in almost any branch of medicine or surgery, or hospitals or nursing to find reference to the leading authorities. On the whole, it may be said to be a useful book of reference, quite apart from its immediate connection with Lewis's Library, whose name has long been a household word to those whose work brings them in daily contact with medical books. The price of the volume is 12s. 6d.—to subscribers to the library 6s.

